# In-situ formation of co particles encapsulated by graphene layers

**DOI:** 10.1186/s42649-022-00076-z

**Published:** 2022-07-14

**Authors:** Minjeong Lee, Gyutae Kim, Gyu Hyun Jeong, Aram Yoon, Zonghoon Lee, Gyeong Hee Ryu

**Affiliations:** 1 School of Materials Science and Engineering, Gyeongsang National University, Jinju, 52828 Republic of Korea; 2 Department of Materials Science and Engineering, Ulsan National Institute of Science and Technology (UNIST), Ulsan, 44919 Republic of Korea; 3 Center for Multidimensional Carbon Materials, Institute for Basic Science (IBS), Ulsan, 44919 Republic of Korea

**Keywords:** Co particle, Encapsulation, Graphene, Co (OH)_2_, STEM

## Abstract

The process of encapsulating cobalt nanoparticles using a graphene layer is mainly direct pyrolysis. The encapsulation structure of hybrids prepared in this way improves the catalyst stability, which greatly reduces the leaching of non-metals and prevents metal nanoparticles from growing beyond a certain size. In this study, cobalt particles surrounded by graphene layers were formed by increasing the temperature in a transmission electron microscope, and they were analyzed using scanning transmission electron microscopy (STEM). Synthesized cobalt hydroxide nanosheets were used to obtain cobalt particles using an in-situ heating holder inside a TEM column. The cobalt nanoparticles are surrounded by layers of graphene, and the number of layers increases as the temperature increases. The interlayer spacing of the graphene layers was also investigated using atomic imaging. The success achieved in the encapsulation of metallic nanoparticles in graphene layers paves the way for the design of highly active and reusable heterogeneous catalysts for more challenging molecules.

## Introduction

Metallic catalysts play a dominant role in industrial applications and the development of catalysts using base metals (Jagadeesh et al. 2013a; Meffere et al. 2015; Jagadeesh et al. 2013b; Czaplik et al. 2007; Zhang et al. 2013) is prevalent because of their distinct electronic structures (Friedfeld et al. 2013) and magnetic properties. In addition, a series of novel heterogeneous catalyst systems using noble metal catalysts (Rahi et al. 2012; Le et al. 2013; Ren et al. 2012; Yan et al. 2013; Ge et al. 2013) have been developed, but noble metals have major drawbacks such as lack of selectivity and low resistance to functional groups (Corma et al. 2008).

In recent years, multi-metal catalysts made by bonding different transition metals have come into prominence (Sammis et al. 2004; Toyofuku et al. 2008; Hashmi et al. 2009; Chinchilla et al. 2007). The driving force behind these efforts is the discovery of more efficient approaches for the synthesis of complex molecules with superior chemical and stereoselectivity that are not accessible through the use of monospecific catalyst systems. The development of these catalysts maximized compatibility while exploiting the benefits of catalysis. Furthermore, transition metals are predominantly applied to find more valuable chemical transformations. This growing interest has led to advances in the field focusing on how the reactivity of transition metal catalysts can be tuned. In addition to this, a strategy has been derived to create metal nanoparticles encapsulated in polymorphic carbon shells (Yao et al. 2014; Galakhov et al. 2010; Liu et al. 2011). The main advantage of encapsulated structures is the ability to tune the electronic structure of metal nanoparticles and tightly control the aggregation of nanoparticles (Chen et al. 2014; Tian et al. 2015). In addition, encapsulation of metal nanoparticles within a porous carbon shell allows for easy access to the catalytically active sites and greatly inhibits mass transfer restrictions (Wu et al. 2014).

Recently, Co encapsulated in a carbon matrix has been developed for various reactions such as catalytic hydrogenation and ORR (Wei et al. 2016; Liu et al. 2015; Yang et al. 2018; Wei et al. 2015). Hybrid Co particles specifically designed to be encapsulated in a carbon material serve as an efficient, selective, and potent catalyst. The interface between the encapsulated Co particles and the graphene layer determines the structural and chemical properties. For metal/graphene systems, interfaces have also been focused on applying graphene to electronic devices where graphene is in contact with metal electrodes and wires (Rosei et al. 1984; Nagashima et al. 1994; Gamo et al. 1997; Abild-Pedersen et al. 2006; Wang et al. 2007; Gruneis et al. 2008). Because these Co/graphene interfaces are two-dimensional internal structures, transmission electron microscopy (TEM) is the most useful method to investigate them. Herein, we report the formation of Co particles encapsulated by graphene layers, which were induced using an in-situ TEM heating holder in a TEM column. We used synthesized Co (OH)_2_ nanosheets and converted them into Co particles. The carbon matrix, which remained amorphous, was transformed into graphene layers surrounding the Co particles at high temperatures (over 800 °C). Interestingly, the number of graphene layers increases when heated to above 1000 °C. The whole process was analyzed using high-resolution STEM.

## Results and discussion

**Fig. 1:**
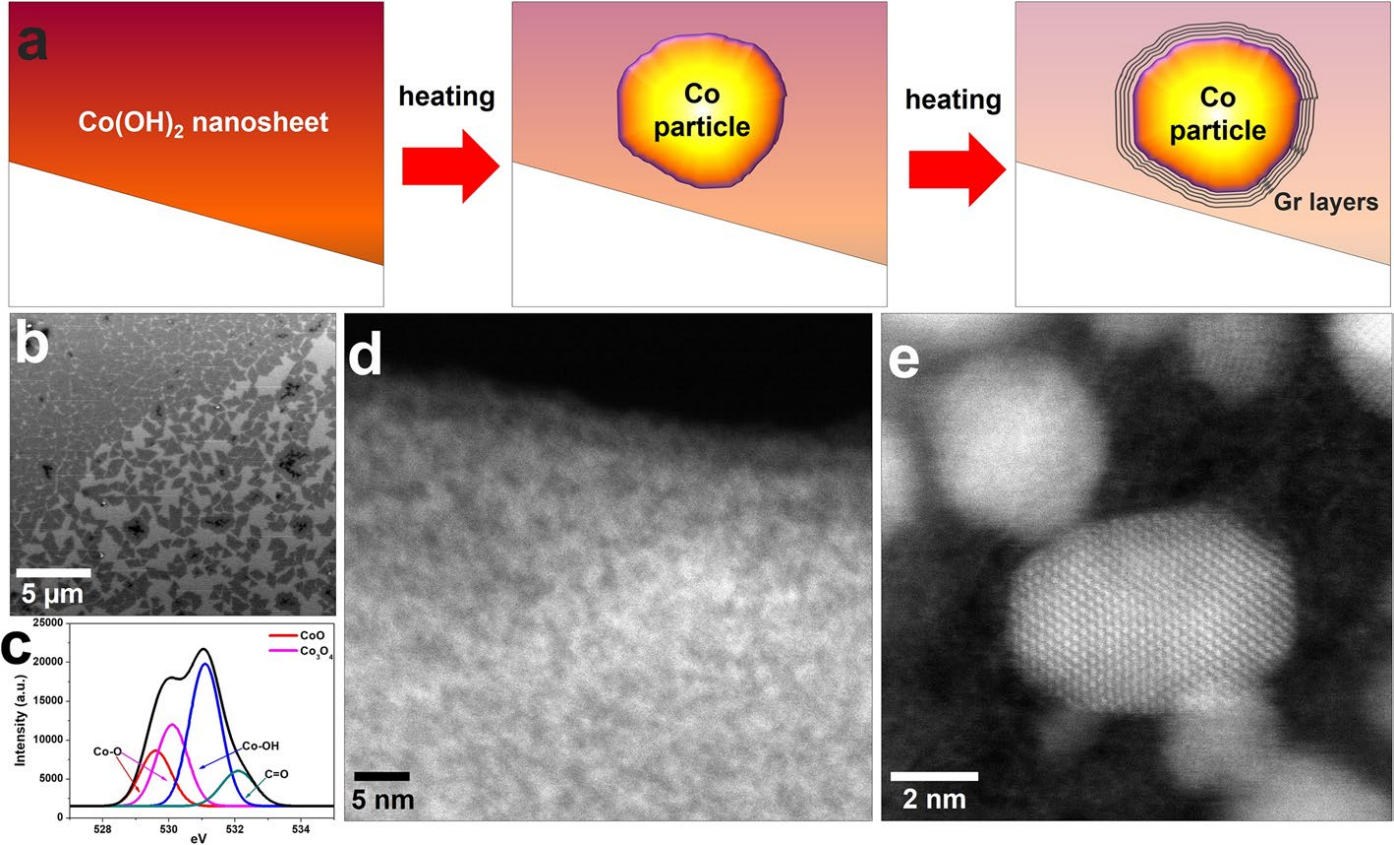
**a** Simple schematic of the whole process. **b** and **c** SEM image and XPS spectra of the synthesized Co (OH)_2_ nanosheets. **d** and **e** ADF-STEM images showing the nanosheet and Co particles formed using a heating holder

Figure 1a shows a encapsulation process of co particle when we have experiment using Co (OH)_2_ nanosheets. We deal with a detailed explanation of the process sequentially. Cobalt hydroxide can be synthesized as nanosheets as shown in Fig. 1b. Their chemical bonding states were confirmed using x-ray photoelectron spectroscopy (XPS) to consist of mainly cobalt hydroxide with some cobalt oxides (Fig. 1c). The peak of O1s at 531.1 eV indicates that the Co atoms are bonded with the OH^−^ group. The deconvolution of O 1 s exhibits two clear peaks located at binding energy at 529.6 eV and 530.5 eV, which is attributed to oxygen in the C-O of CoO crystal and Co_3_O_4_ crystal, respectively (Petitto et al. 2004; Wang et al. 2019). We transferred the specimen onto a heating chip to induce a heating pulse into the specimen. The morphology and structure of the nanosheets were investigated using high-resolution scanning transmission electron microscopy (HR-STEM). Figure 1c shows a high-resolution image of the synthesized nanosheet, which is visualized as an amorphous phase (Fig. 1d). When the heating pulse is applied to them, the amorphous nanosheets transform into crystalline cobalt particles above 500 °C (Fig. 1e).

**Fig. 2:**
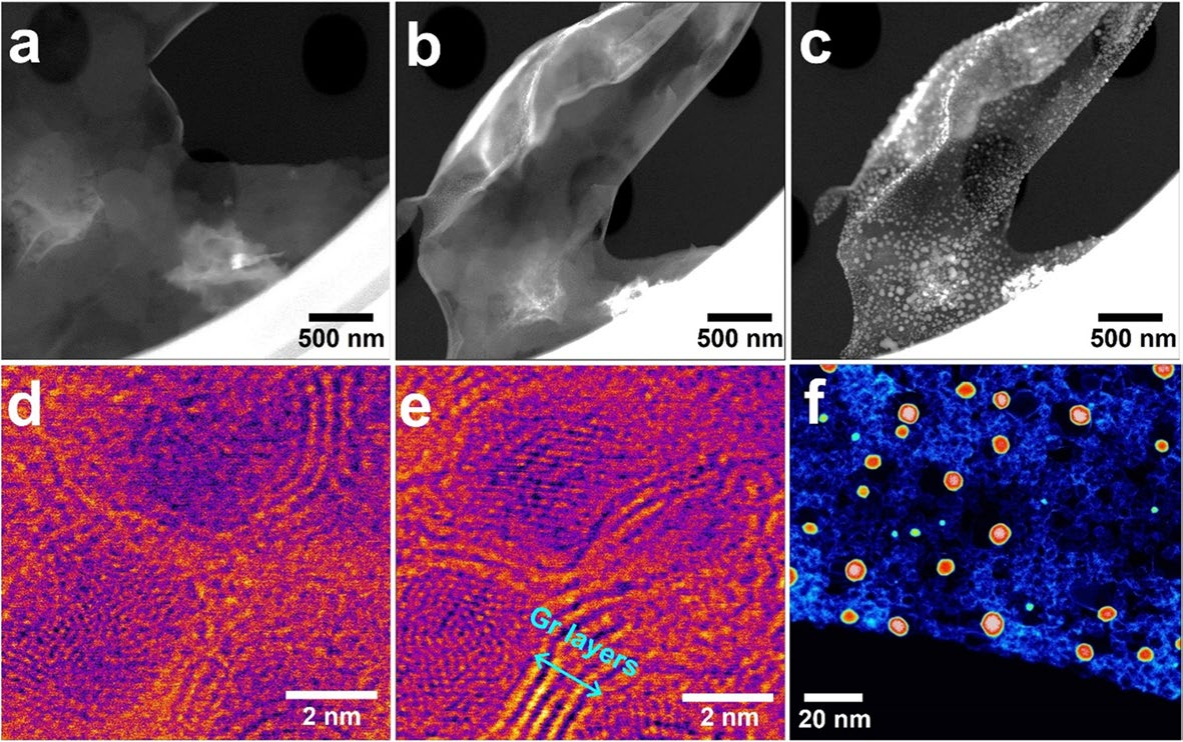
**a**-**c** Successive HAADF-STEM images showing the transformation **d** and **e** BF-STEM images showing the Co particles with thin carbon film at 500 °C and 800 °C at the same position, respectively. **f** Low-scale HAADF-STEM images showing the Co particles with graphene layers

When the temperature increases to 1100 °C, the nanosheets progressively transform as shown in Fig. 2a to c, which are successive high angle annular dark filed (HAADF)-STEM images showing the overall transformation behavior of the sheet in the same region. The transformation initiation is not implemented in only a specific area, but occurs in the entire sheet area to which temperature is applied, and Co atoms constituting Co (OH)_2_ are aggregated to form a large amount of Co particles. When heated to 500 °C, Co particles are formed as shown in Fig. 1, and the hydrocarbons remaining on the surface of the Co (OH)_2_ nanosheet form a thin film as shown in Fig. 2c. Figure 2d shows the bright filed (BF)-STEM image of the Co particles with a thin carbon film at 500 °C. Above 800 °C, the carbon film begins to crystallize gradually, and carbon layers (graphene layers) are formed at the edge of the Co particle (Fig. 2e). The Co particles encapsulated by graphene layers are visualized in Fig. 2f.

**Fig. 3:**
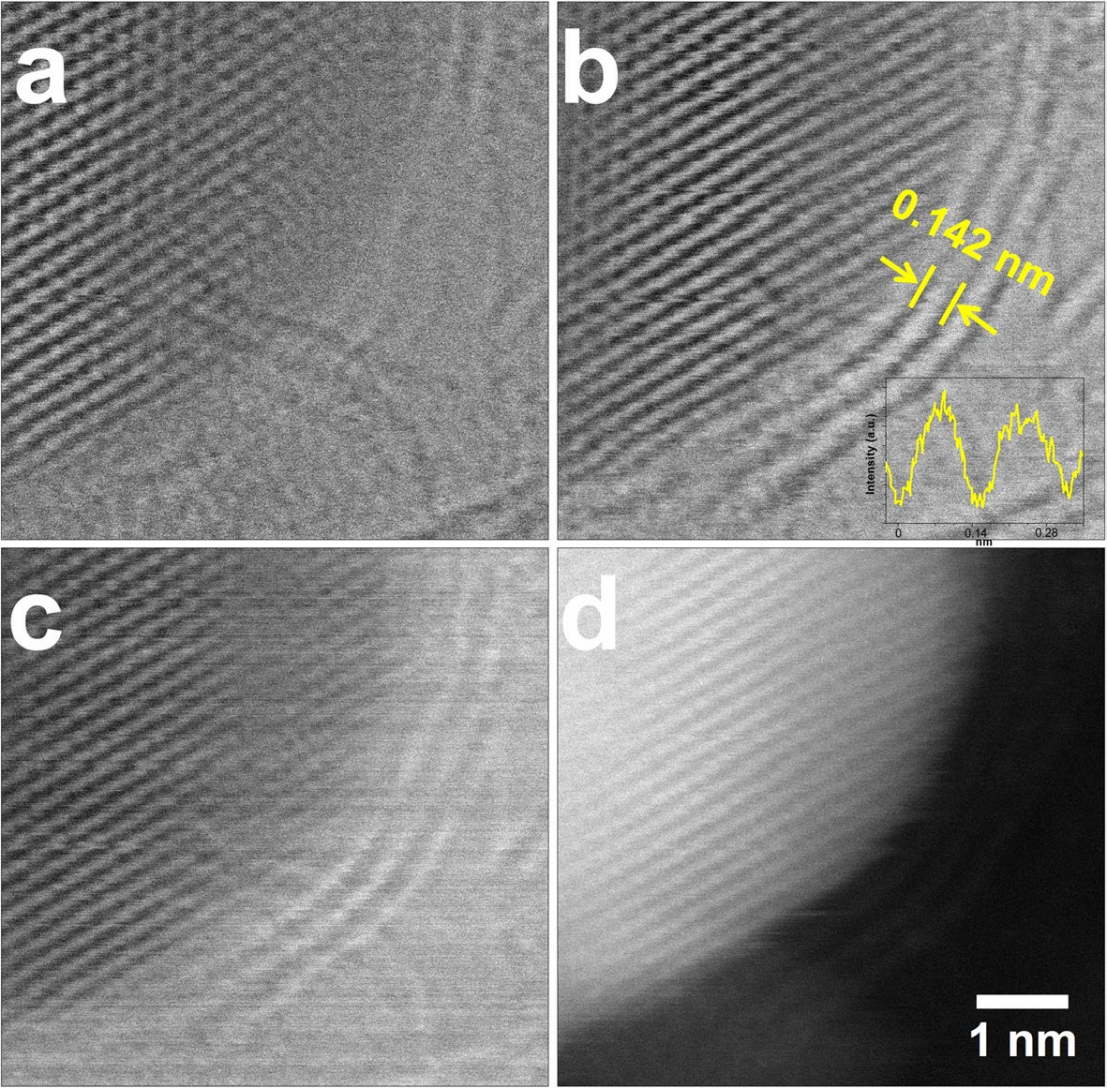
Co particle encapsulated by graphene layers, which collected by (**a**) BF, (**b**) and (**c**) ADF and (**d**) HAADF detectors, respectively

We observed an encapsulated Co particle using STEM mode, which allows us to collect various images using bright field (BF), annular dark field (ADF: DF2, DF4), and HAADF detectors simultaneously. Even if the material is composed of the same element, the degree of visualization differs depending on the type of detector used in STEM mode. This is because the detection degree of the scattered electron beam varies according to the scattering angle of the electron beam as it scatters through the material. Therefore, we obtained BF, DF2, DF4, and HAADF images in STEM mode to investigate the morphology and structure of a Co particle encapsulated by graphene layers as shown in Fig. 3. The distance between the graphene interlayers is measured to be 0.142 nm, which is consistent with the graphene interplanar spacing.

**Fig. 4:**
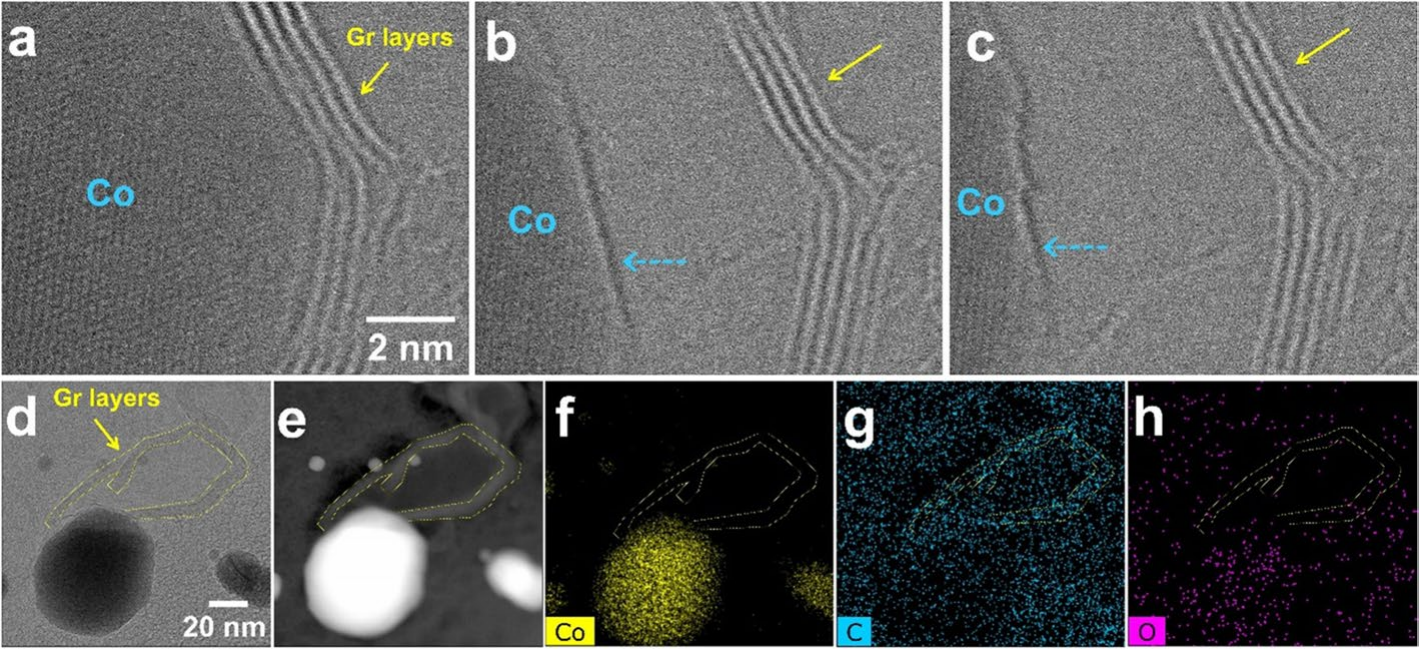
**a**-**c** Successive BF-STEM images showing formation of graphene layers surrounding Co particle. **d**-**e** BF and HAADF-STEM images of Co particle and remaining graphene layers. **f**-**h** EDS mapping of Co, C, and O elements at the same region as (**d**) and (**e**)

The graphene layer forms to surround the Co particles, and even if the Co particles partially move, the graphene layer maintains its shape. As shown in Fig. 4a, four graphene layers surround the Co particles, and they maintain their morphology while the Co particles move in the direction of the light blue arrow (Fig. 4b and c). This result suggests that the graphene layers surrounding the Co particles were not formed temporarily but were formed in a stacked form while maintaining the interlayer spacing with crystallinity. After EDS mapping confirmed the graphene layers remained after the movement of the Co particles, the graphene layers visualized in Fig. 4d and e were also included in the entire supported carbon film region, confirming the overall C mapping in the field of view.

**Fig. 5:**
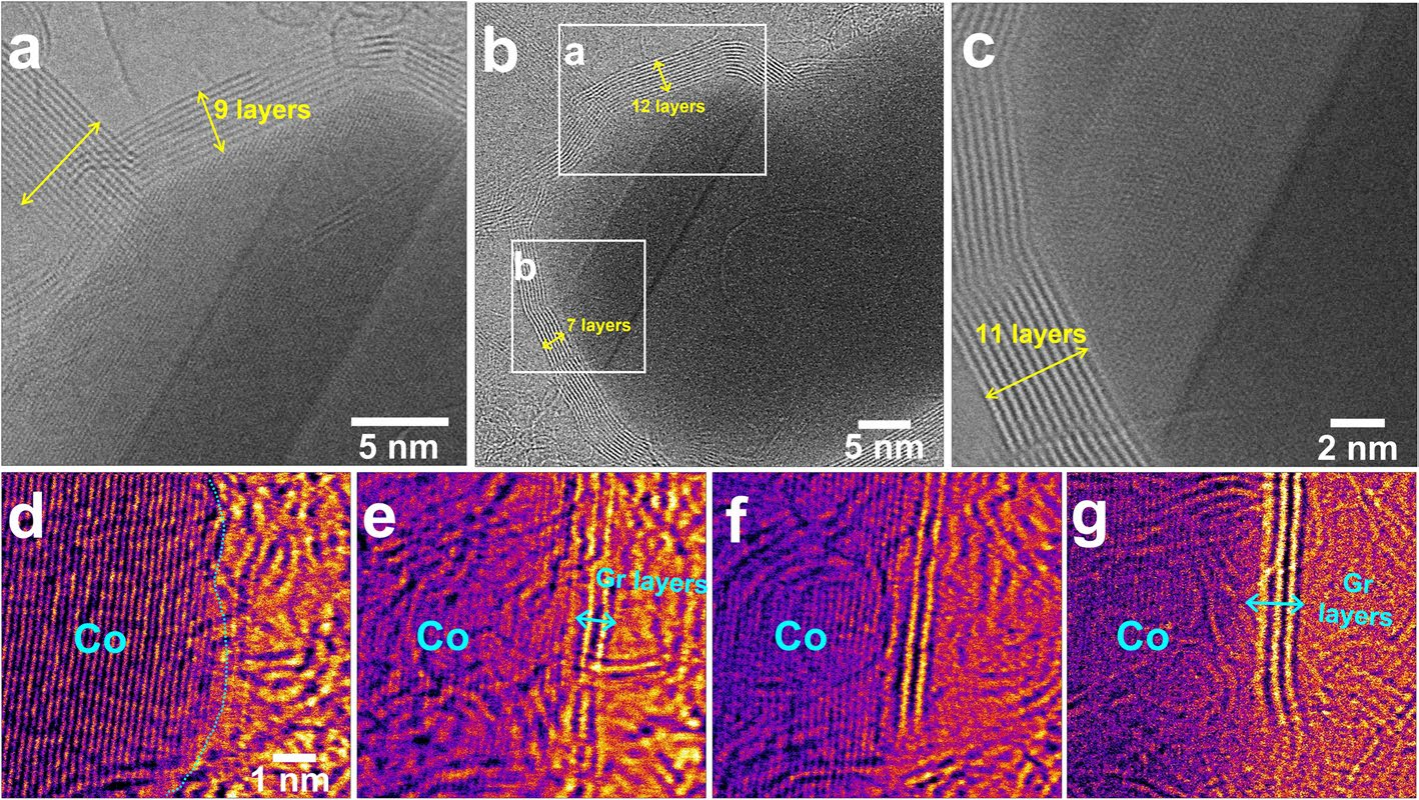
**a**-**c** Successive BF-STEM images showing the growth of graphene layers at 1050 °C. **d**-**g** Successive BF-STEM images showing a initial states on the growth of graphene layers at another position

The graphene layer surrounds the Co particles without a gap between the interface of the Co particles and the graphene layer. The number of graphene layers, which are observed prominently at 800 °C or higher, increases when a heating pulse of 1000 °C or higher is applied. Fig. 5 shows BF-STEM images of the graphene layers identified encapsulating the Co particle at 1050 °C. The number of graphene layers at the top of the Co particle was 9 (Fig. 5a), but when the results of continuous image acquisition were confirmed, the number of layers increased to 12 (Fig. 5b). In addition, the number of graphene layers surrounding the lower left part of the Co particle increased from 7 layers to 11 layers (Fig. 5c). This is because, as in the case of synthesizing graphene using a metal catalyst, Co particles act as a catalyst, and the remaining amorphous carbon source expands the number of graphene layers at 1000 °C or more and is smoothly crystallized into graphene. At another position, the growth process is also observed as shown in Fig. 5d-g.

## Conclusions

We summarize that the behavior of Co particles encapsulated by graphene layers formed by high temperature and electron beam irradiation. The amorphous carbon remaining in the synthesized Co (OH)_2_ nanosheet exists in the form of a thin film, which crystallizes when a temperature of 800 °C or higher is applied. The crystallized carbon grows into a graphene layer surrounding the Co particles, and the number of graphene layers gradually increases due to the catalytic activation of Co at temperatures above 1000 °C. All these behaviors were observed through STEM imaging, and the graphene layer composed of low elements was observed using BF and ADF images in STEM mode, which has the advantage of using various detectors at the same time. The results of this work show a versatile and scalable technique that can be used to fabricate structured graphene materials.

## Methods

### Synthesis

Co (OH)_2_ nanosheets were synthesized using the aqueous nutrient solution containing 2 mM cobalt nitrate hexahydrate and 2 mM hexamethylenetetramine (HMTA). Depending on the opening area of a container, a calculated amount of chloroform solution of sodium hexadecyl sulfate (SHS) was added to the water-air interface. After about 30 minutes, the container was capped and placed in a convection oven at 70 °C for typically 180 minutes. The synthesized Co (OH)_2_ sheets were scooped using an TEM grid for imaging.

### Transmission Electron microscopy

STEM images were acquired using an aberration-corrected FEI Titan Cubed TEM (FEI Titan3 G2 60–300), which was operated at a 200 kV acceleration voltage with a monochromator. Dose rate was 72.5 A/m^2^.

## Data Availability

The datasets used and/or analyzed during the study are available from the corresponding author on reasonable request.

## Abbreviations

XPS: X-ray Photoelectron Spectroscopy
SEM: Scanning Electron Microscopy
HAADF-STEM: High Angle Annular Dark Field Scanning Transition Electron Microscopy
ADF: Annular Dark Field
BF: Bright Field
DF: Dark Field
